# Healing wounds, defeating biofilms: *Lactiplantibacillus plantarum* in tackling MRSA infections

**DOI:** 10.3389/fmicb.2023.1284195

**Published:** 2023-12-05

**Authors:** Ashish Kumar Dubey, Mohini Sharma, Parul, Sachin Raut, Pawan Gupta, Neeraj Khatri

**Affiliations:** ^1^IMTech Centre for Animal Resources & Experimentation (iCARE), CSIR-Institute of Microbial Technology (IMTECH), Chandigarh, India; ^2^Academy of Scientific and Innovative Research (AcSIR), Ghaziabad, Uttar Pradesh, India; ^3^Department of Molecular Biology, CSIR-Institute of Microbial Technology (IMTECH), Chandigarh, India

**Keywords:** MRSA, AMR, probiotics, wound healing, *Lactoplantibacillus plantarum*, biofilm, cell-free supernatant

## Abstract

**Introduction:**

Methicillin-resistant *Staphylococcus aureus* (MRSA) infections are well-known hospital-borne infections and are a major contributing factor to global health concerns of antimicrobial resistance due to the formation of biofilms. Probiotics are known to assist in the healing of wounds through immunomodulation and also possess anti-pathogen properties via competitive inhibition. The probiotic bacterium, *Lactiplantibacillus plantarum* MTCC 2621 and its cell-free supernatant (Lp2621) have previously been reported to have antibacterial, excellent antioxidant, and wound healing activity in *in vitro* conditions and wounds contaminated with *S. aureus* in mice.

**Methods:**

In the current study, we evaluated its anti-MRSA, biofilm inhibition and eradication efficacy, immunomodulatory activity in THP-1 cells, and wound healing potential in wounds contaminated with MRSA infection in mice.

**Results:**

In agar well diffusion assay, Lp2621 showed anti-MRSA activity and revealed dose-dependent inhibition and eradication of biofilm by crystal violet assay as well as by Confocal Scanning Laser Microscopy (CLSM) analysis. Further, Lp2621 showed immunomodulatory activity at varied concentrations as measured by IL-6 and IL-10 gene expression in THP-1 cells. Similar findings were observed in serum samples of mice after treatment of excision wound contaminated with MRSA infection by Lp2621 gel, as evident by expression of IL-6 (pro-inflammatory) and IL-10 (anti-inflammatory) cytokines.

**Conclusions:**

Overall, our results show that Lp2621 has potent anti-MRSA and antioxidant properties and can prevent and eliminate biofilm formation. It also showed promise when applied to mice with MRSA-infected wounds.

## Graphical Abstract

Schematic representation of Lp2621 as an inhibitor of MRSA: *in vitro* and *in vivo* studies. 
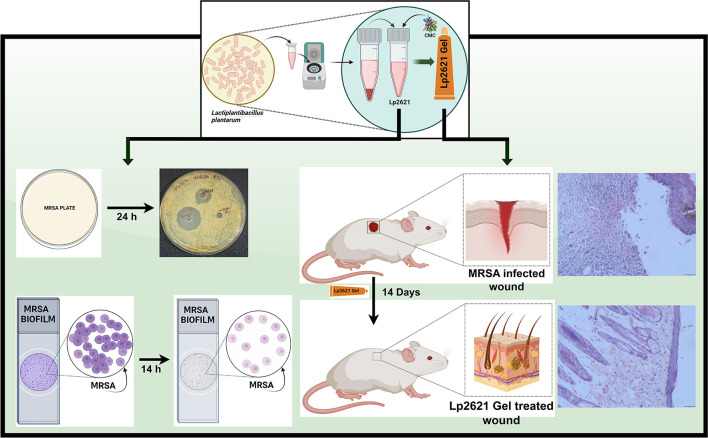

## 1 Introduction

Wound healing is a multi-faceted biological phenomenon that allows the body to repair damaged tissue. Cells (keratinocytes, fibroblasts, and macrophages) are directed to the site of the wound as a result of an inflammatory response and initiate the healing process through interaction with stem cells. This leads to a variety of biochemical processes that allow the body to repair physical tissue (Eming et al., 2014). Wounds are most commonly infected with *Staphylococcus aureus* (Tong et al., 2015), which can develop resistance to methicillin and antibiotics (lactam) through the manifestation of a foreign penicillin-binding protein (PBP, PBP2a) (Stapleton and Taylor, 2002).

MRSA is a grave public health problem worldwide, and infections caused by these germs are among the most difficult therapeutic challenges. Infections caused by bacterial biofilms are becoming more widespread around the world. Bacteria in biofilms are frequently resistant to standard antibiotic therapy because, over time, they have developed a number of defensive mechanisms against conventional antibiotic therapy (Xiu et al., 2021). All MRSA strains are capable of microplate attachment and biofilm formation. Developing biofilms and antibiotic resistance are crucial to the success of *S. aureus* pathogens in both hospitals and community surroundings (Akbari-Ayezloy et al., 2017). Methicillin-resistant *Staphylococcus aureus* (HA-MRSA & CA-MRSA) are hospital- and community-associated infections and are additional categories for MRSA infections that can affect different parts of the body and vary in severity from minor skin contaminations to more lethal infections such as bacteremia, skin and soft tissue infections, bone and joint infections, endocarditis, pneumonia, and sepsis (Siddiqui and Koirala, 2022). Approximately 20–50 cases/100,000 population for *S. aureus* bacteremia are reported annually, which alone causes more causality than AIDS, TB, and viral hepatitis combined (Hal et al., 2012).

MRSA has emerged in recent years primarily as an isolated bacterium in wound cultures (Naylor et al., 2001; Scriven et al., 2003; Reddy et al., 2007) that can dramatically slow wound healing and increase the risk of complications (Thimmappa et al., 2021). First, the inflammation caused by the bacteria can damage healthy tissue and impede the healing process. Second, MRSA can form a biofilm on the site that protects the germs from the body's immune response and makes it difficult for antibiotics to reach and destroy the germs (Simonetti et al., 2022). Treatment of MRSA-infected wounds usually involves a combination of antibiotics and wound care. Vancomycin and other MRSA-fighting antibiotics may be prescribed to treat the infection (Cong et al., 2020).

To effectively treat wounds and infections, a variety of biological strategies have been developed and adopted. These include the use of various scaffolds, carriers, and patches derived from algae, as well as stimuli-responsive hydrogels and ceria (CeO_2_)-modified nanoparticles (Zhong et al., 2021; Li et al., 2022; Ma et al., 2022). Only a small number of antibiofilm strategies are being used in clinical settings, and the rest still need to be developed significantly. The use of nanotechnology-based treatments to fight bacterial biofilm infections has been suggested by Xiu et al. (2021).

Probiotics improve health through oral or topical administration and may assist in the reduction of the risk of MRSA infection and improve outcomes for MRSA-infected patients (Cella et al., 2023). *S. aureus* and clinical MRSA isolates are prevented from growing *in vitro* by a wide range of lactobacilli and bifidobacteria strains (Sikorska and Smoragiewicz, 2013). However, to fully assess the potential benefits of probiotics in preventing or treating MRSA infections, more research is warranted. Probiotics are most likely to be ineffective in neonates and/or in individuals with certain clinical problems such as cancer, diabetes mellitus, leaky gut syndrome, and convalescence after organ transplantation (Kothari et al., 2019). Probiotic strains may have antibacterial activity against MRSA. One study found that a probiotic strain called *Lactobacillus reuteri* suppressed the growth of MRSA *in vitro* (Prince et al., 2012), while *Lactobacillus plantarum* prevented the spread of MRSA in mice (Sikorska and Smoragiewicz, 2013). The bioactive molecules present in the cell-free supernatant of lactic acid bacteria exhibited anti-staphylococcal effects by upregulating β-defensin and modulating cytokines and chemokines during wound healing phases (Ong et al., 2020). Hydrogels loaded with *Lactobacillus rhamnosus* (HPF@L.rha) significantly decreased infection and inflammation, encouraged the production of new collagen and epithelium, and accelerated wound healing (Mei et al., 2022). Mice given curcumin-loaded solid lipid nanoparticles (CSLNs) along with a probiotic dressing (*L. plantarum* UBLP-40) demonstrated faster wound closure and less bioburden in the wound area. Through immunomodulation, antibacterial activity, suppression of pathogenic toxins, and anti-inflammatory attributes, these therapies also aided in the promotion of wound healing (Sandhu et al., 2023).

Previously, the cell-free supernatant of *Lactiplantibacillus plantarum* MTCC 2621 from our lab demonstrated the antimicrobial activity, antioxidant properties, and wound-healing potential of Lp2621 (Dubey et al., 2021). In this study, we sought to evaluate anti-MRSA, radical scavenging, biofilm inhibition, and eradication activities of Lp2621. We also provide evidence showing that Lp2621 has the potential to heal excisional wounds infected with MRSA 831 (Graphical abstract).

## 2 Materials and methods

### 2.1 Materials and reagents

Nutrient agar, MRS broth and agar, and Mueller Hinton Broth (MHB) were purchased from HiMedia. TRIzol reagent was acquired from Invitrogen. 2,2-Diphenyl-1-picrylhydrazyl (DPPH), ascorbic acid, and 4α-phorbol 12-myristate 13-acetate-PMA were bought from Sigma Aldrich. BD enhanced sensitivity kit (IL-6, Cat No. 562236 and IL-10, Cat No. 562263) was purchased from BD Biosciences.

### 2.2 Culture of *L. plantarum* MTCC 2621 and preparation of Lp2621

*L. plantarum* MTCC 2621 was obtained from the Microbial Type Culture Collection (MTCC) (CSIR-IMTECH). The MRSA 831, a clinical strain, was a gift from Dr. Hemraj Nandanwar's laboratory at CSIR-IMTECH. The MRS broth was used to culture the *L. plantarum* at 37°C, and the grown culture was centrifuged (5,000 rpm, 10 min) for collection of cell free supernatant (CFS) and stored at 4°C and filtered through a 0.22 μm filter for animal tissue culture experimentation. The MRSA strain was cultured in Mueller Hinton Broth (MHB). The Institutional Biosafety Committee approved the use of MRSA 831 in experiments.

**Figure 1:**
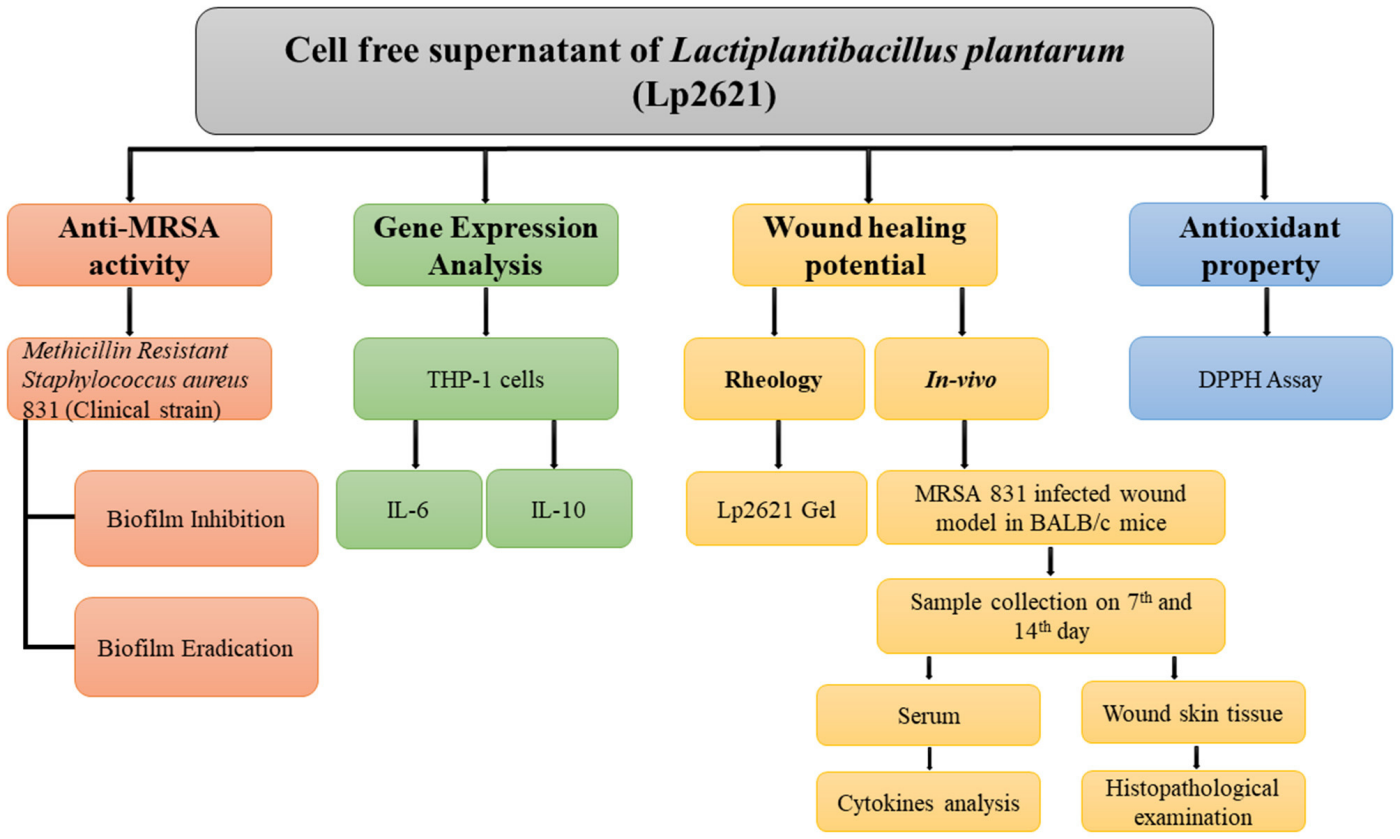
Flowchart outlining the research plan for examining Lp2621′s ability to treat MRSA-infected wounds and to suppress, eliminate, and eradicate biofilms.

The experimental design of the study is illustrated in a flowchart in Figure 1.

### 2.3 Anti-MRSA activity of Lp2621

The antibacterial activity against MRSA 831 was evaluated using the agar well diffusion technique. The MRSA strain was inoculated on the nutrient agar (10^6^ CFU/ml). Six mm diameter wells were punched with a sterile well borer, and 80 μL of Lp2621 was added into the test well. Vancomycin (positive control) and MRS broth (negative control) were used and incubated at 37°C. The inhibitory zone diameter was calculated with a ruler after 24 h (Dahiya and Purkayastha, 2012).

### 2.4 Biofilm inhibition and eradication activity of Lp2621

The method of Yang et al. (2021) was used to test whether LP2621 could inhibit the formation and eradication of MRSA-induced biofilms (Yang et al., 2021). Briefly, MRSA 831 overnight culture was suspended in MHB, 5 × 10^5^ CFU/ml were inoculated in 96-well plates, and Lp2621 was incubated for 24 h at 37°C. The media used was taken out, and the biofilms were rinsed three times with 1X PBS and fixed with 99% v/v methanol for 15 min at room temperature. Crystal violet 0.1% w/v was applied to stain the biofilm for 10 min at room temperature in the dark.


(1) $$\begin{array}{l} \text{Biofilm inhibition }\left(\%\right)\text{ =}\\ \;\left(\frac{\text{Control absorbance }-\text{ Test absorbance}}{\text{Control absorbance}}\right)\times \text{ 100} \end{array}$$

The method was followed with some changes used by Perumal and Mahmud (2013) for the biofilm eradication study of Lp2621. Briefly, MRSA831 overnight culture was inoculated at a 96-well plate and kept for biofilm production at 37°C for 48 h. 1X PBS (pH 7.4) was used to wash the wells. The established biofilm was treated with Lp2621 and incubated at 37°C for 24 h. The media used were taken out, and the biofilms were rinsed three times with 1X PBS, and 99% v/v methanol was used for fixation for 15 min at 25°C. Crystal violet 0.1% w/v was applied to fixed biofilm for 10 min at 25°C and kept in the dark.


(2) $$\begin{array}{l} \text{Biofilm eradication }\left(\%\right)\text{=}\\ \;\left(\frac{\text{Control absorbance }-\text{Test absorbance}}{\text{Control absorbance}}\right)\times \text{ 100} \end{array}$$

#### 2.4.1 Confocal scanning laser microscopy analysis

Confocal microscopy was used to visualize the MRSA-formed biofilm morphology. MRSA 831 was cultured overnight in MHB at a concentration of 5 × 10^5^ CFU/ml (0.01 OD). The culture was then inoculated onto poly L-lysine coated coverslip into a 6-well plate and supplemented with different dilutions of Lp2621, i.e., 50, 12.5, and 0.78% (v/v) for 12–14 h. The medium was removed, and the biofilms were washed with 1X PBS (pH 7.4) three times and stained with 3 μM SYTO9 for 15–20 min at room temperature in the dark. The coverslips were washed after staining with 1X PBS (pH-7.4) three times and placed onto a slide (on 10 μL live antifade reagent added), and mounted onto sides. The prepared slides were then visualized under confocal scanning laser microscopy at 60X (oil) magnification.

### 2.5 Antioxidant properties

DPPH assay is a reliable and accurate method to assess the ability of antioxidants to scavenge free radicals. We followed the DPPH method of free radical scavenging activity for evaluating the antioxidant potential of Lp2621 used by Sancineto et al. (2016) with slight modifications (Sancineto et al., 2016). In brief, the CFS was diluted two-fold in methanol up to 0.78% v/v and mixed with a methanol solution of DPPH (25 μg/ml). The reaction was allowed for 15 min, 30 min, and 24 h in the dark at room temperature. Ascorbic acid (AA) (60 μg/ml) was used as a positive standard, while the control had DPPH radical and methanol solution. The sample and methanol combination were used as sample blank.

The percentage inhibition of DPPH was calculated using the following equation:


(3) $$\begin{array}{l} \%\;of\;Inhibition\;of\;the\;DPPH\;radical=\\ \left[Abs\left(control\right)-\left(\frac{Abs\left(sample\right)-Abs\left(blank\right)}{Abs\left(control\right)}\right)\right]\times \;100 \end{array}$$

Abs (control) = The absorbance of DPPH and methanol.

Abs (sample) = The absorbance of sample.

Abs (blank) = The absorbance of the sample and methanol.

### 2.6 Gene expression analysis of IL-6 and IL-10

#### 2.6.1 THP-1 cell cultivation

THP-1 (human leukemia monocyte cell line T-helper) cells were revived using RPMI 1640 medium (Gibco), with 10% FBS (Gibco), and antimicrobial 1% penicillin-streptomycin (Gibco) and maintained in CO_2_ incubator at 37°C and 5% CO_2_. Cells were plated in a 6-well culture plate (10^6^ cells/ml) and incubated with PMA (4-phorbol 12-myristate 13-acetate) (20 ng/ml) for 24 h at 37°C and 5% CO_2_ in a CO_2_ incubator for differentiation to macrophages. The differentiated THP-1 cells were treated with different concentrations of Lp2621, and RNA was extracted from the treated cells for analysis of gene expression of IL-6 and IL-10 by qRT-PCR.

#### 2.6.2 Extraction of RNA and cDNA synthesis

TRIzol was used to extract total RNA from the cells. Concentration and purity of RNA were checked on a TECAN UV-VIS spectrophotometer (Nanodrop Technologies, Thermo Fisher Scientific), and the cDNA was made using reverse transcription with a high-capacity cDNA synthesis kit (Applied Biosystems cat no. 4368814). The qRT-PCR was carried out on a qTOWER3G (Analytik Jena). The primers used for different genes have been given in Table 1. GAPDH, the housekeeping gene, was used as an internal control. The mRNA level in each sample was determined using the 2^−Δ*ΔCt*^ technique.

**Table 1:** Primer's sequence of genes.

**Gene**	**Forward (5^′^-3^′^)**	**Reverse (3^′^-5^′^)**
IL-6	AGCCACTCACCTCTTCAGAAC	GCCTCTTTGCTGCTTTCACAC
IL-10	GTGATGCCCCAAGCTGAGA	CACGGCCTTGCTCTTGTTTT
GAPDH	TGCACCACCAACTGCTTAGC	GGCATGGACTGTGGTCATGAG

### 2.7 Rheological properties of Lp2621 gel

The bacterial culture of *L. plantarum* 2621 containing 1 × 10^9^ CFU/ml was centrifuged for 10 min at 5,000 rpm, and the cell-free supernatant (CFS) was taken. A total of 2% carboxy methyl cellulose (CMC) was added to CFS and mixed continuously until a homogenous gel of Lp2621 was formed. The gel was kept at 4°C for subsequent use. Rheological properties of the gel were conducted utilizing a rheometer (MCR 102, Anton Paar) equipped with a Peltier plate temperature-controlled device for precise thermoregulation. A parallel plate (40 mm) with a set gap of 1.0 mm was used to study the viscosity flow curve under the shear range between 0.001–1,000 s^−1^ to determine the flow behavior of the gel (Srivastava and Choudhury, 2021).

### 2.8 Lp2621 gel improved healing of wounds contaminated with MRSA infection in mice

The Institutional Animal Ethics Committee of CSIR-Institute of Microbial Technology (CSIR-IMTECH) approved the use of animals (IAEC/22/06). Experiments on mice were conducted as per the guiding principles of the Committee for the Control and Supervision of Experiments on Animals (CCSEA), Ministry of Fisheries, Animal Husbandry, and Dairying, India.

BALB/c mice (8 weeks) were obtained from the IMTECH Centre for Animal Resources and Experimentation (iCARE) of CSIR-IMTECH. Mice were housed in IVC cages in the ABSL-2 facility of iCARE, and pelleted food and water were provided *ad libitum*. Mice were randomly assigned to different groups before the experiment for a period of 1 week to acclimatize. In our previous study, we showed the beneficial effect of Lp2621 on normal wounds as well as wounds contaminated with *S. aureus* infection (Dubey et al., 2021).

Since contamination of wounds with MRSA is difficult to treat, we next pondered whether Lp2621 would be effective in the treatment of such wounds. We, therefore, designed an experiment wherein excision wounds were created in mice, and the wounds were contaminated with MRSA infection, followed by treatment with Lp2621 gel. In brief, mice were divided into the following five groups (*n* = 8 mice per group): negative (only wound), disease (wound + infection), vehicle (wound + infection + CMC), positive (wound + infection + betadine), and Lp2621 group (wound + infection + Lp2621). The mice were acclimatized for a week, anesthetized, dorsal hair removed, wiped, and disinfected (70% ethanol). The previous method for the creation of a wound was followed (Dubey et al., 2021). The wound was infected with 1 × 10^5^ CFU/ml of MRSA 831 (Hoffmann et al., 2020) with slight modification, and each group of mice was topically treated with respective treatment for up to 21 days (twice a day) after 24 h of infection. On days 0, 7, 14, and 21, images of wounds were taken by DSLR camera and processed using ImageJ software for wound area calculation.


(4) $$\text{Percent}\;\text{wound}\;\text{contraction}\;=\;\frac{\text{Healed}\;\text{area}}{\text{Total}\;\text{area}}\times \;\text{100}$$

The wound tissues and blood samples were collected from different groups of mice on days 7 and 14 for histopathological examination and cytokine analysis, respectively. The tissues were fixed in 10% neutral buffered formalin, cleaned in xylene, dehydrated with graded alcohol, molded in paraffin, and sections of 4–5 μm thickness were prepared and stained with hematoxylin and eosin (H&E) and were then examined under a light microscope.

The serum samples were used for immunomodulatory analysis. Cytokine analysis was performed using a BD multiplex CBA kit through FACS.

### 2.9 Statistical analysis

The statistical analysis was done using a one-way analysis of variance (ANOVA). The results are expressed as the mean ± SD.

## 3 Results

### 3.1 Anti-MRSA activity of Lp2621

**Figure 2:**
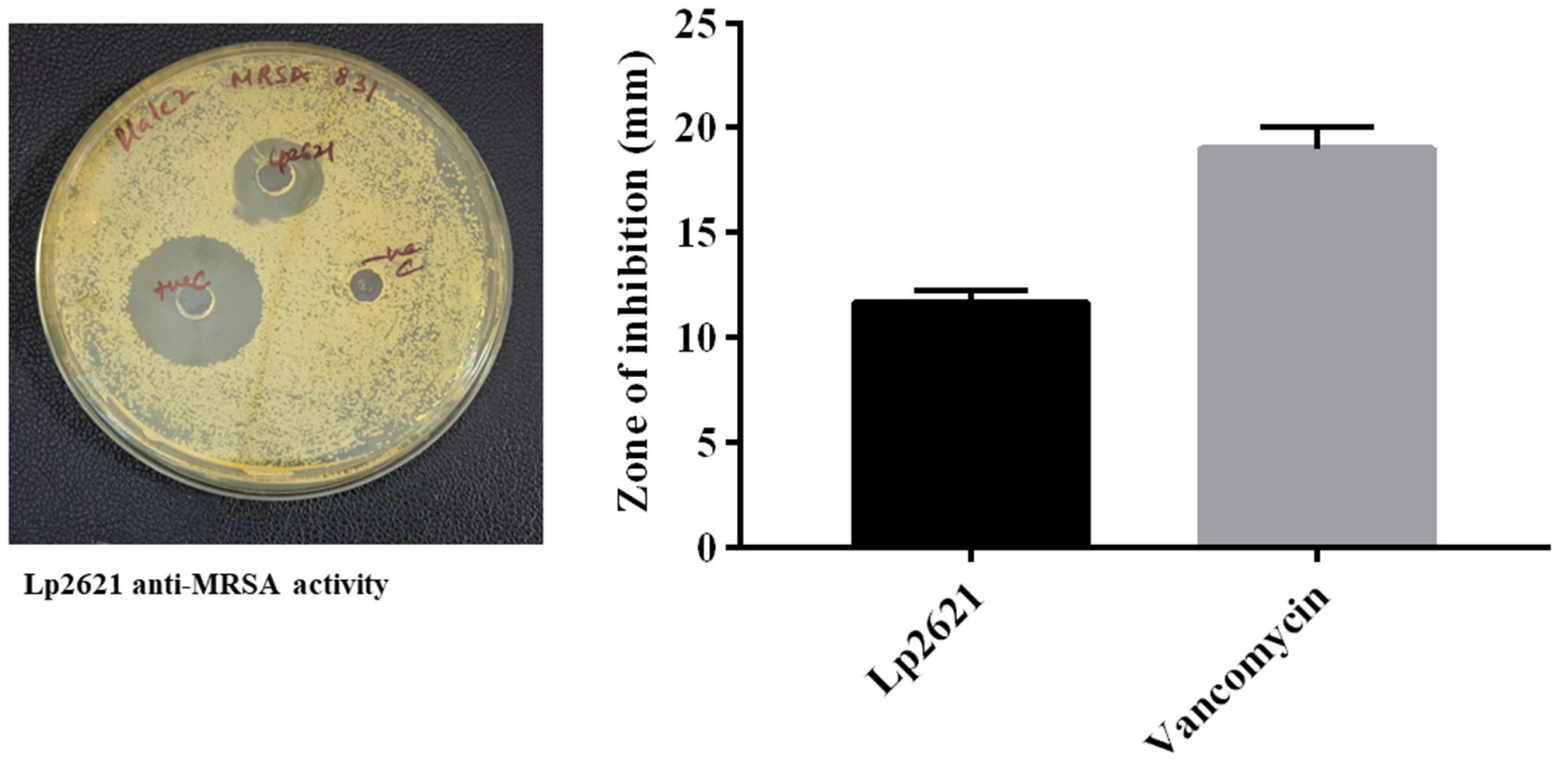
Agar well diffusion technique to measure Lp2621′s effectiveness against MRSA 831. Data from three separate biological investigations were presented in triplicate.

Lp2621 exhibited anti-MRSA activity in agar well diffusion assay. The measured zone of inhibition for Lp2621 was 11.66 ± 0.57 mm, and for vancomycin (positive control), it was 19.00 ± 1.00 mm (Figure 2).

### 3.2 MRSA biofilm inhibition and eradication activity of Lp2621

**Figure 3:**
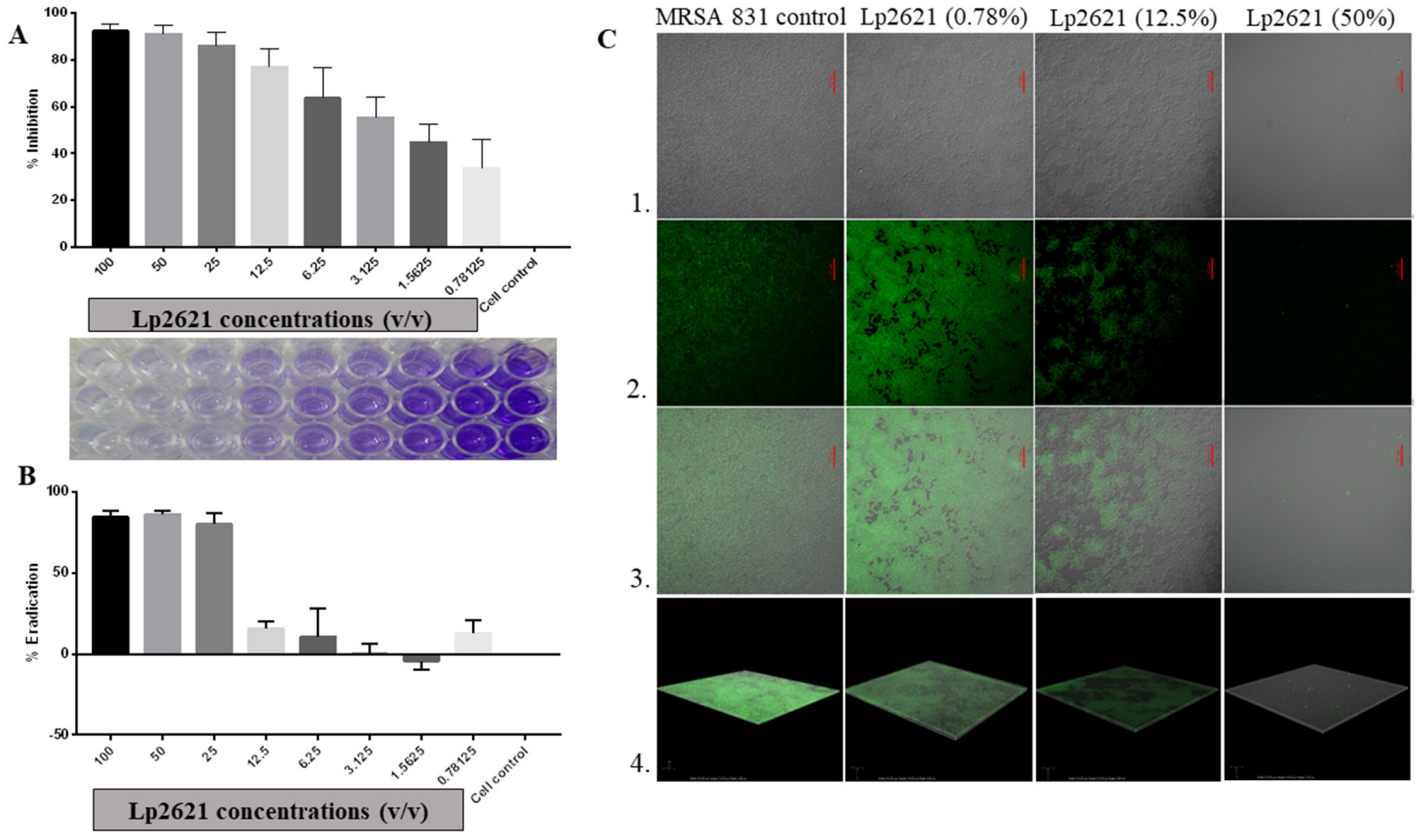
The effect of Lp2621 on biofilm: insights into inhibition and eradication; **(A)** Effective inhibition of biofilm formation; **(B)** Eradication of MRSA 831 by Lp2621; **(C)** Confocal laser scanning microscopy (CLSM) - Series 1, 2, 3, and 4 show TD, dye only, merged image of TD and dye, and Z-stack images, respectively. SYTO-9 (3μM) dye was used for observation at a wavelength of 488 and magnification of × 60 (oil). Scale bar: 25 μm. The data shown are from three independent biological experiments (mean ± SD).

The underlying mechanisms controlling the biofilm formation of MRSA strains in various types of human infections are not very clear. Lp2621 could inhibit biofilm formed by MRSA in a dose-dependent manner (Figure 3A). At higher concentrations (100–12.5% v/v), it showed significantly higher inhibition of 77%, whereas the inhibition was 33% at lower concentrations (0.78% v/v). Moreover, higher concentrations of Lp2621 (100, 50, and 25% v/v) were found to be highly effective in eradicating and removing the biofilm formed by MRSA 831 (>80%) (Figure 3B). To further investigate the antibiofilm effect of Lp2621 against MRSA 831 and observe the morphological changes in biofilm, CLSM was performed. CLSM images (Figure 3C) showed thick biofilms in MRSA 831 containing live cells; however, the thickness and viability of cells significantly decreased after treatment with different concentrations of Lp2621 (dose-dependent). The biofilm was almost completely inhibited at a concentration of 50% v/v of Lp2621.

### 3.3 Antioxidant properties of Lp2621

**Figure 4:**
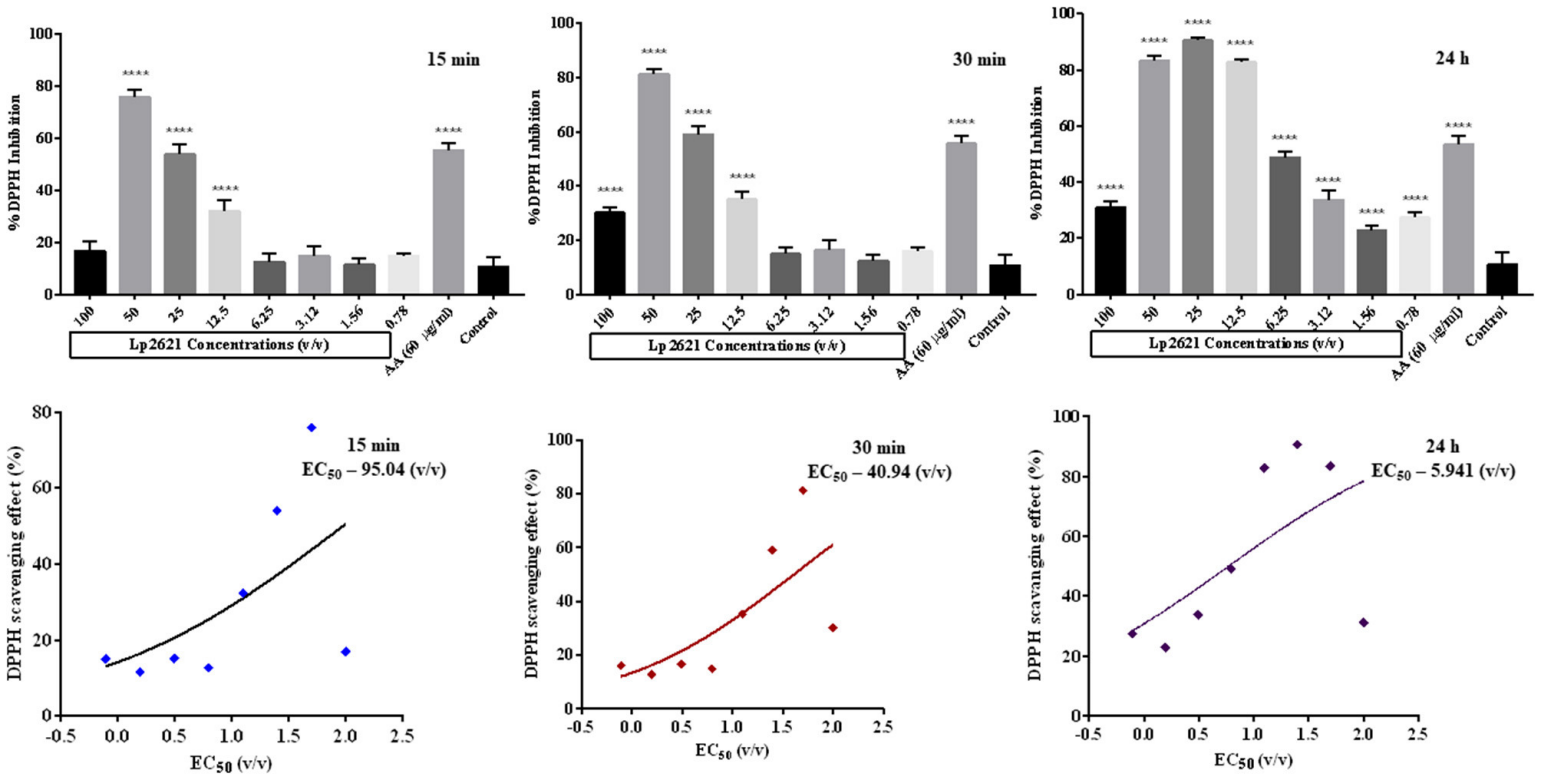
Percent DPPH inhibition (Lp2621: 100 to 0.78% v/v) and EC_50_ values of DPPH scavenging activity of Lp2621. Data presented are in triplicate from three independent biological experiments (mean ± SD). ****mean *p* < 0.0001.

Having observed the anti-MRSA effect of the cell-free supernatant, we next planned to explore the antioxidant potential of Lp2621 using a DPPH assay. Ascorbic acid was taken as a positive standard (60 μg/ml). Figure 4 shows the scavenging activity of Lp2621 for free radicals. DPPH inhibition mediated by Lp2621 was highly significant at higher concentrations (50, 25, and 12.5 v/v) at 15 and 30 min compared to the control (methyl alcohol and DPPH). At a concentration of 50% v/v, Lp2621 showed 76 and 81% inhibition, respectively, after 15 and 30 min of reaction time (*p* < 0.0001). Interestingly, after 24 h of reaction, Lp2621 exhibited better scavenging activity at various concentrations, and maximum inhibition was 90% at 25% v/v (*p* < 0.0001). The effective concentration (EC_50_) was found to be 95.04 v/v (at 15 min), 40.94 v/v (at 30 min), and 5.94 v/v (at 24 h) at respective time points.

### 3.4 Modulation of relative gene expression of IL-6 and IL-10 by Lp2621

**Figure 5:**
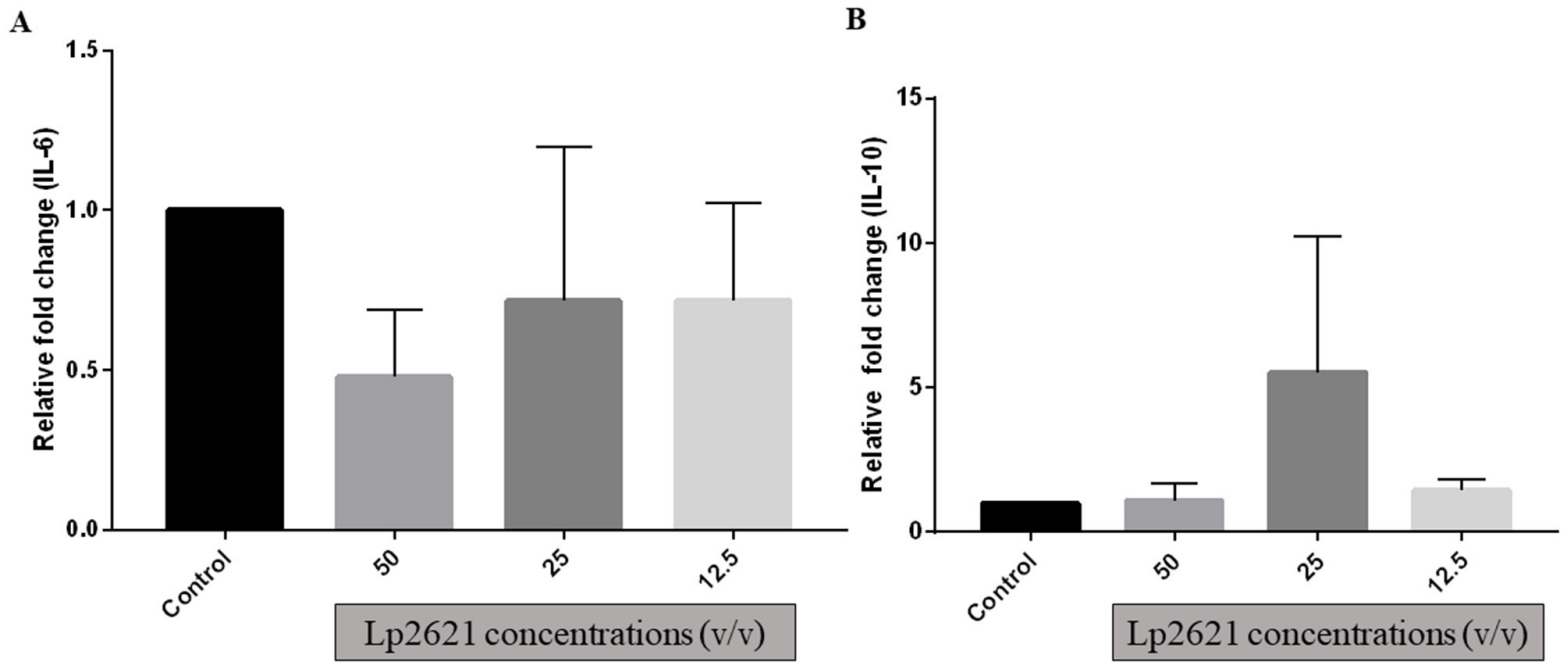
Gene expression analysis **(A)** IL−6 and **(B)** IL−10 genes in THP-1 cells treated with different concentrations of Lp2621. Fold change was compared with control (untreated cells) and calculated with respect to GAPDH (glyceraldehyde-3-phosphate dehydrogenase). Data are representative of three independent experiments in triplicate, expressed as mean ± SD.

THP-1 cells are the most commonly used cells for immune response (inflammation) studies. The uniform genetic basis of THP-1 minimizes the degree of variability in cell phenotype, which increases the reproducibility of results. qRT-PCR was used to verify gene expression of IL-6 and IL-10 after incubating differentiated THP-1 cells with various concentrations of Lp2621 for 24 h. Although there were changes in mRNA expression of IL-10 (increased at 25% v/v) and IL-6 (decreased at various concentrations 12.5–50% v/v) after treatment with Lp2621 as compared to the control gene, i.e., GAPDH, these changes in expression levels were statistically non-significant (Figure 5).

### 3.5 Rheological profile of Lp2621 gel

**Figure 6:**
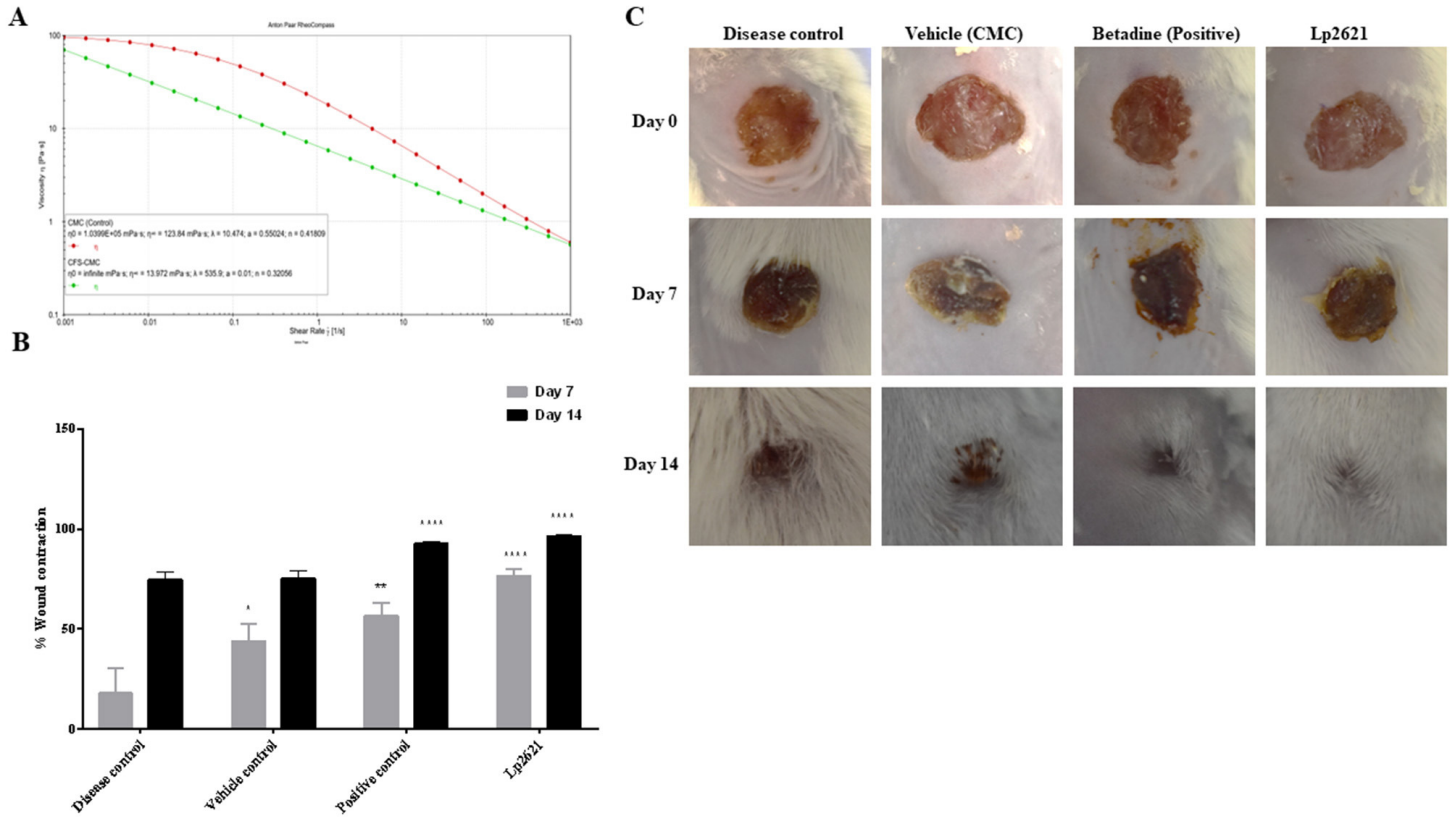
Rheological profile of Lp2621 gel **(A)** and its effect on wound healing; **(B)** percent wound contraction in wounds of different groups of mice; and **(C)** representative images of wound healing in mice. Data are presented as mean ± SD. *p = 0.02, **p = 0.001, and ****p < 0.0001.

The flow behavior of the Lp2621 gel was determined by rheological tests. From the rheological data (Figure 6A), it was found that the viscosity decreased with increasing shear rate, indicating that it was a pseudoplastic, non-Newtonian material. Here, the viscosities at zero (η0) and at infinite shear rate (η∞) were determined by fitting the Carreau-Yasuda model. The η∞ values for CMC were 123.84 mPa·s and for CFS-CMC 13.972 mPa·s, showing that the viscosity of the gel decreased, which is desirable for topical applications.

### 3.6 Lp2621 gel improved healing of wounds contaminated with MRSA infection in mice

MRSA infections are difficult to treat as these bugs are resistant to most antibiotics. Therefore, we wanted to investigate whether treating wounds contaminated with MRSA with Lp2621 could shorten wound healing time. Topical application of Lp2621 gel resulted in a recovery of wound infected with MRSA. Faster wound healing was observed in mice treated with Lp2621 and betadine on day 7 as compared to CMC (vehicle) treated and the diseased control (*p* < 0.0001) untreated mice, and at day 14, wound healing was statistically more significant in Lp2621 and betadine treated mice than other groups. This is evident by an increase in the percent wound contraction in mice treated with our gel and betadine (Figures 6B, C).

#### 3.6.1 Histopathological examination

**Figure 7:**
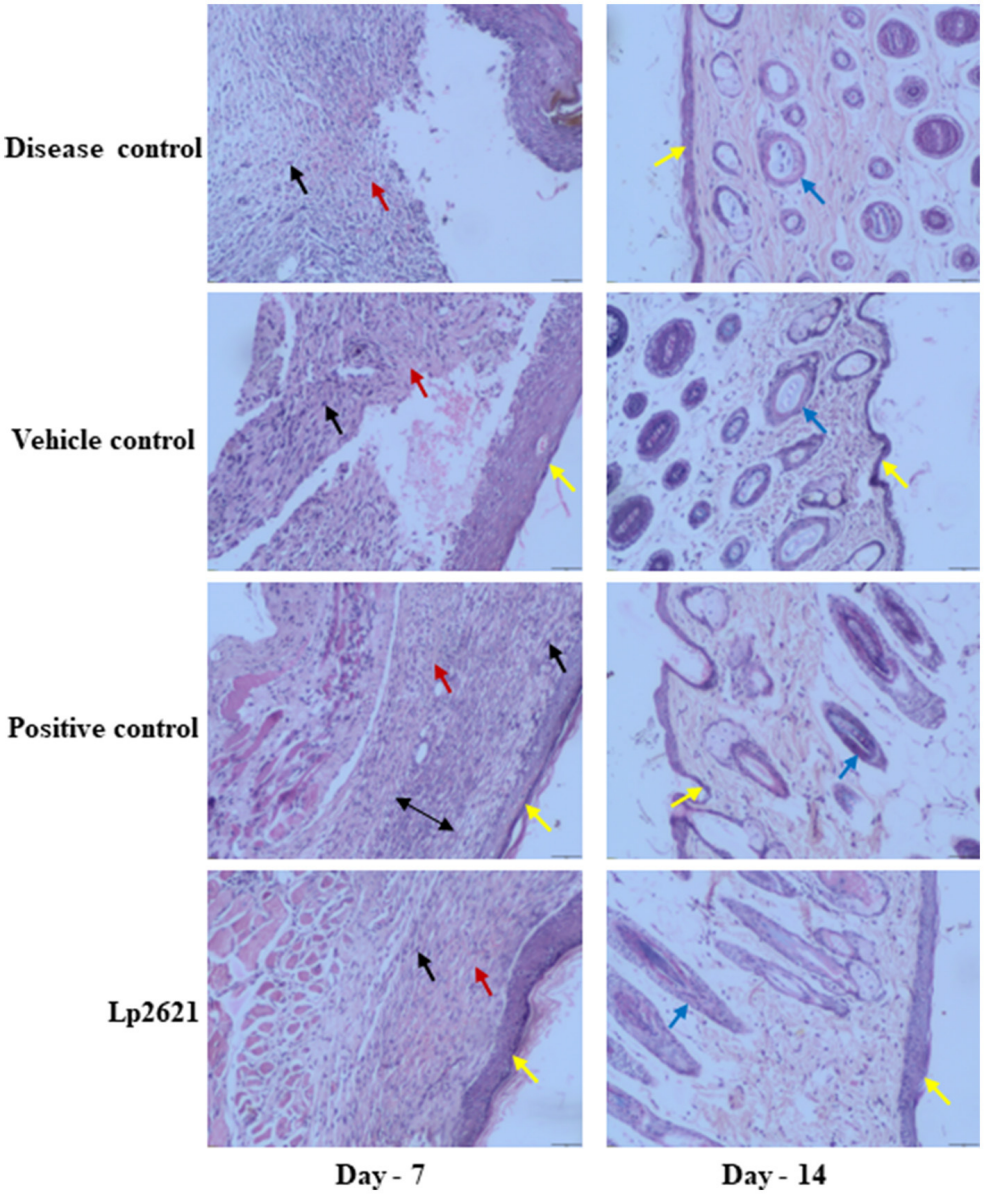
Representative histopathological images of wounds of various groups at days 7 and 14 (scale-50 μm). Re-epithelization (yellow), vascularization (red), fibroblasts (black), deposition of collagen (double-sided arrow), and follicle formation (blue arrow).

The results of histopathologic evaluation of the wound tissues of mice from each treatment group on days 7 and 14 after wounding are shown in Figure 7. The results revealed pronounced infiltration of polymorphonuclear leukocyte (PMNL) in disease control group mice at day 7. Vehicle-treated group showed PMNL infiltration, fibroblast proliferation, vascularization, and some epithelialization (day 7, vehicle control), whereas mice treated with Lp2621 and betadine showed significant recovery of wound as indicated by mild PMNL infiltration, marked fibroblast proliferation, vascularization, and a distinct and thicker epidermis at day 7. Day 14 data showed the development of capillaries, fibroblasts, collagen, and connective tissue in the treated groups (betadine and Lp2621), whereas fibroblast cell formation, granulation tissue, follicle formation, and finally, re-epithelialization of the wound skin were delayed in the control groups (diseased and treated with vehicle).

#### 3.6.2 Immunomodulatory potential of Lp2621

**Figure 8:**
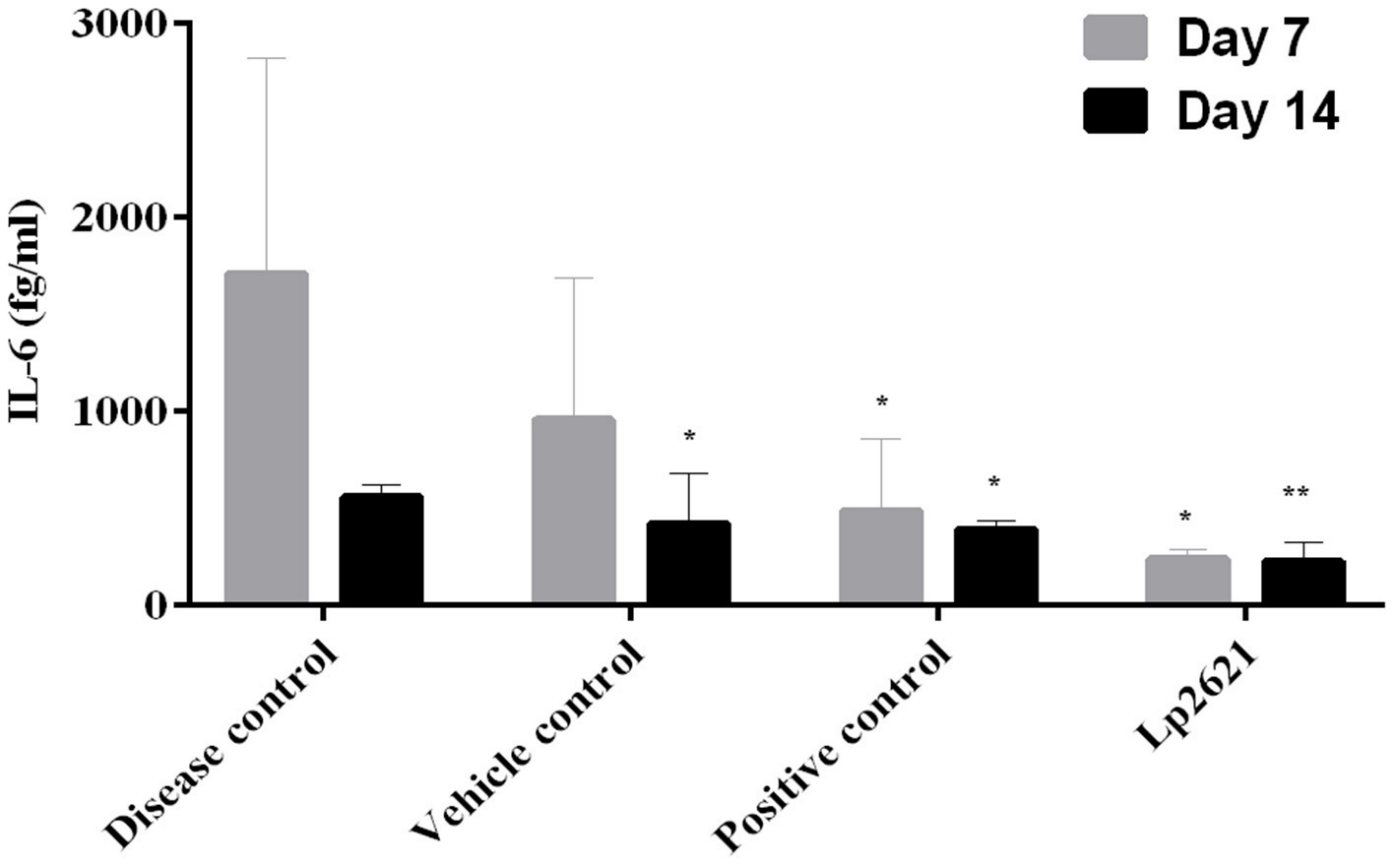
Immunomodulatory activities of Lp2621. Analysis of cytokine immune response profile in response to Lp2621 treatment in serum of mice. Data are expressed as mean ± SD. *p = 0.04 and **p = 0.03.

The immunomodulatory effect of Lp2621 was studied in the serum of mice. Quantification of IL-6 was performed using the BD Enhanced Sensitivity Kit according to the manufacturer's instructions. Levels of IL-6 (pro-inflammatory cytokine) were higher in disease-control mice on day 7 and decreased significantly after treatment with betadine and Lp2621 on days 7 and 14. However, this difference between the vehicle- and betadine-treated groups was not significant at day 7 but was significant at day 14. Interestingly, we observed a significant decrease in the levels of IL-6 in the Lp2621-treated mice compared with the vehicle-treated (^**^p = 0.03) mice on both day 7 and day 14 (Figure 8). However, we could observe IL-10 expression only in a few samples of the betadine and Lp2621-treated groups of mice at day 14. Therefore, no conclusion can be drawn from this observation (data not shown).

## 4 Discussion

The present study was envisaged to study the ability of Lp2621, CFS from *L. plantarum*, to inhibit MRSA infection. Furthermore, the free radical scavenging activity (measured by the DPPH method), immunomodulatory analysis, and healing of excision wounds in mice models infected with MRSA were also established. Earlier studies have shown that *L. plantarum* MTCC 2621 and its cell-free supernatant have antibacterial activity against *Staphylococcus aureus* MTCC 737, *Micrococcus luteus* MTCC 106, *Klebsiella pneumonia* MTCC 618, *Pseudomonas aeruginosa* MTCC 1934, *Bacillus subtilis* MTCC 441, and *Escherichia coli* MTCC 739 (Sreevani and Kumari, 2013; Dubey et al., 2021). This prompted us to investigate the anti-MRSA role of Lp2621. We observed significant antibacterial activity of Lp2621 against the clinical strain MRSA 831 using an agar gel diffusion assay. Further, we were interested in checking the anti-biofilm activity of Lp2621 because biofilm helps these bugs in their survival against many antibiotics. Biofilms are aggregates of complicated, sessile populations of microorganisms that are either firmly rooted in an extracellular matrix or attached to a surface. As a result, the bacteria are resistant to adverse environments and antibacterial treatments, making effective treatment extremely difficult (Roy et al., 2018). Lactic acid bacteria (LAB) and their metabolites, viz. exopolysaccharides, enzymes, peptides, polyphenols, antibiotics, and lantibiotics-nisin have been investigated as possible biofilm biocontrol agents (Parisot et al., 2008; Roy et al., 2018; Song et al., 2020). Given that Lp2621 exhibited anti-MRSA activity and that MRSA is known to form biofilms, we next used confocal microscopy to test the effectiveness of Lp2621 in preventing and eradicating biofilm. In the biofilm inhibition assay, cells were exposed to Lp2621 at varying concentrations, including 50, 12.5, and 0.78% v/v, against MRSA 831. The findings revealed that biofilm formation was totally suppressed at these concentrations. Karska-Wysocki et al. (2010) discovered the antibacterial activity of *Lactobacillus acidophilus* and *Lactobacillus casei*, which contain antimicrobial components that can limit the growth and destroy MRSA cells. These observations are in agreement with earlier reports utilizing CFS of *Lactobacillus brevis* KCCM 202399 and *L. fermentum* TCUESC01 (Melo et al., 2016; Kim et al., 2022). Lp2621 has been shown to limit the amount of biofilm that pathogenic clinical MRSA strains may form, making it a possible candidate for use in the fight against antimicrobial resistance (AMR). Experiments on biofilm eradication have demonstrated how challenging it is to entirely eliminate a biofilm. *L. plantarum* and its by-products have been shown to possess a high capacity to block the infection-causing abilities of *P. aeruginosa* and MRSA (Onbas et al., 2019; Moghadam et al., 2020). Our findings also suggest that Lp2621 has strong biofilm inhibition activity against clinical isolate of MRSA 831 at higher doses (Figure 3).

Excellent DPPH scavenging activity was demonstrated by Lp2621, which has previously been noted as the cell-free supernatant of various lactobacilli in the literature (Xing et al., 2015; Sornsenee et al., 2021). Next, we evaluated the immunomodulatory activity of Lp2621 and found that it has the potential to alter the expression of pro- and anti-inflammatory cytokines, according to cytokine gene expression study and quantification by qRT-PCR using THP-1 cells. These results corroborated with previously published studies (Bermudez-Brito et al., 2013; Marco et al., 2018). To further validate the role of Lp2621 in the treatment of wounds infected with MRSA in mice, a gel was prepared having CFS of *L. plantarum* MTCC 2621. The rheological properties of the gel were evaluated in terms of viscosity, and the gel was found to be suitable for use (non-Newtonian pseudoplastic). The gel showed tremendous healing activity in wounds infected with MRSA. *L. plantarum* applied to a wound before and after the initiation of MRSA infection reduced the occurrence of MRSA superinfection in a burn wound model in rats (Sürmeli et al., 2019). Previous studies have also shown that probiotics/lactobacilli play a role in healing various types of wounds, such as oral wounds (Han et al., 2019), excisional wounds in Wistar rats (Sinha et al., 2019), chronic ischemic wound lesions (von Ossowski et al., 2013), and burn wounds (Moghadam et al., 2020).

Histopathological analysis of the skin tissue revealed that Lp2621 had an impact on the lesions, as evidenced by PMNL infiltration, fibroblast proliferation, vascularization, and epidermal development. Similar histological alterations, including PMNL infiltration, collagen deposition, angiogenesis, and tissue granulation, were seen in an excisional wound treated with a cell-free supernatant of *Bifidobacterium bifidum* in conjunction with chitosan (Bazjou et al., 2022). *L. rhamnosus*, when administered orally to Swiss mice, stimulated epithelization, reduced macrophage and mast cell infiltration, improved angiogenesis and blood circulation, collagen deposition and scarring, and also reduced inflammation and fibrogenesis in wounded skin (Moreira et al., 2021).

Another key finding of our study is that Lp2621 treatment of mice with wounds contaminated with MRSA resulted in a decreased expression of IL-6 in serum. These observations are in agreement with our previous studies, where decreased IL-6 levels were reported in mice with wounds infected by *S. aureus* following treatment with Lp2621 (Dubey et al., 2021). Probiotic bacterial strains and/lactic acid bacteria have been shown in numerous studies (Karamese et al., 2016; Johnson et al., 2020; Dubey et al., 2021) to reverse histopathological abnormalities in wounds and modify pro- and anti-inflammatory cytokines in serum. These findings are the first tangible proof that Lp2621 promotes wound healing by limiting the growth of the clinical MRSA strain *in vivo*.

In summary, our results show that Lp2621, a probiotic CFS, has potent antibacterial and antioxidant properties. It also exhibits *in vitro* biofilm inhibition and eradication activity, as well as anti-MRSA activity. Additionally, it showed promise in the treatment of mice with MRSA-infected wounds. Our findings support the notion that *L. plantarum* produces metabolites capable of suppressing growth and eradicating MRSA. Although more research is needed to explore the mechanistic insights and characterize the metabolites responsible for these activities, the strategy of using Lp2621 as a therapeutic intervention against MRSA and other antibiotic-resistant microbiological infections can be weighed.

## Data availability statement

The original contributions presented in the study are included in the article/supplementary material, further inquiries can be directed to the corresponding author.

## Ethics statement

Institutional Animal Ethics Committee (IAEC) of CSIR-Institute of Microbial Technology, Chandigarh has reviewed and approved the animal study protocol (No. IAEC/22/06). The study was conducted in accordance with the local legislation and institutional requirements.

## Author contributions

AKD: Data curation, Formal analysis, Investigation, Methodology, Validation, Writing—original draft, Writing—review & editing. MS: Methodology, Writing—review & editing. P: Methodology, Writing—review & editing. SR: Formal analysis, Writing—review & editing. PG: Formal analysis, Writing—review & editing. NK: Conceptualization, Data curation, Formal analysis, Funding acquisition, Project administration, Resources, Supervision, Visualization, Writing—original draft, Writing—review & editing.

## References

[B1] Akbari-Ayezloy E. Hosseini-Jazani N. Yousefi S. Habibi N. (2017). Eradication of methicillin resistant S. *aureus* biofilm by the combined use of fosfomycin and β-chloro-L-alanine. Iranian J. Microbiol. 9, 1. PMC5533998 28775817

[B2] Bazjou A. Jafari P. Marjani A. Akbari N. (2022). Effect of cell-free supernatant of bifidobacterium bifidum combined with chitosan biodegradable film on full thickness wound healing in rats. Physiol. Pharmacol. 26, 468–479. 10.52547/phypha.26.4.2

[B3] Bermudez-Brito M. Muñoz-Quezada S. Gomez-Llorente C. Matencio E. Bernal M. J. Romero F.. (2013). Cell-free culture supernatant of bifidobacterium breve CNCM I-4035 decreases pro-inflammatory cytokines in human dendritic cells challenged with salmonella typhi through TLR activation. PLoS ONE 8, 59370. 10.1371/journal.pone.0059370 PMC3595273 23555025

[B4] Cella M. A. Coulson T. MacEachern S. Badr S. Ahmadi A. Tabatabaei M. S.. (2023). Probiotic disruption of quorum sensing reduces virulence and increases cefoxitin sensitivity in methicillin-resistant staphylococcus aureus. Scient. Rep. 13, 1–14. 10.1038/s41598-023-31474-2 PMC10020441 36928453

[B5] Cong Y. Yang S. Rao X. (2020). Vancomycin resistant staphylococcus aureus infections: a review of case updating and clinical features. J. Adv. Res. 21, 169–176. 10.1016/j.jare.2019.10.005 32071785 PMC7015472

[B6] Dahiya P. Purkayastha S. (2012). Phytochemical screening and antimicrobial activity of some medicinal plants against multi-drug resistant bacteria from clinical isolates. Indian J. Pharmac. Sci. 74, 443–450. 10.4103/0250-474X.108420 PMC3660871 23716873

[B7] Dubey A. K. Podia M. Priyanka Raut S. Singh S. Pinnaka A. K.. (2021). Insight into the beneficial role of *Lactiplantibacillus plantarum* supernatant against bacterial infections, oxidative stress, and wound healing in A549 cells and BALB/c mice. Front. Pharmacol. 12, 728614. 10.3389/fphar.2021.728614 34803678 PMC8600115

[B8] Eming S. A. Martin P. Tomic-Canic M. (2014). Wound repair and regeneration: mechanisms, signaling, and translation. Sci. Transl. Med. 6, 265sr6. 10.1126/scitranslmed.3009337 PMC4973620 25473038

[B9] Hal S. J. Jensen S. O. Vaska V. L. Espedido B. A. Paterson D. L. Gosbell I. B. (2012). Predictors of mortality in Staphylococcus aureus bacteremia. Clin. Microbiol. Rev. 25, 362–386. 10.1128/CMR.05022-11 22491776 PMC3346297

[B10] Han N. Jia L. Su Y. Du J. Guo L. Luo Z.. (2019). *Lactobacillus reuteri* extracts promoted wound healing via PI3K/AKT/β-Catenin/TGFβ1 pathway. Stem Cell Res. Ther. 10, 1–11. 10.1186/s13287-019-1324-8 31391121 PMC6686392

[B11] Hoffmann J. P. Friedman J. K. Wang Y. McLachlan J. B. Sammarco M. C. Morici L. A.. (2020). In situ treatment with novel microbiocide inhibits methicillin resistant staphylococcus aureus in a murine wound infection model. Front. Microbiol. 10, 3106. 10.3389/fmicb.2019.03106 32038549 PMC6990143

[B12] Johnson B. Z. Stevenson A. W. Prêle C. M. Fear M. W. Wood F. M. (2020). The role of IL-6 in skin fibrosis and cutaneous wound healing. Biomedicines 8, 101. 10.3390/biomedicines8050101 32365896 PMC7277690

[B13] Karamese M. Aydin H. Sengul E. Gelen V. Sevim C. Ustek D.. (2016). The immunostimulatory effect of lactic acid bacteria in a rat model. Iranian J. Immunol. 13, 220–228. 10.22034/iji.2016.33410 27671513

[B14] Karska-Wysocki B. Bazo M. Smoragiewicz W. (2010). Antibacterial activity of *Lactobacillus acidophilus* and *Lactobacillus casei* against methicillin-resistant Staphylococcus aureus (MRSA). Microbiol. Res. 165, 674–686. 10.1016/j.micres.2009.11.008 20116228

[B15] Kim J. H. Jang H. J. Lee N-K. Paik H-D. (2022). Antibacterial and antibiofilm effect of cell-free supernatant of lactobacillus brevis KCCM 202399 isolated from Korean fermented food against streptococcus Mutans KCTC 5458′. J. Microbiol. Biotechnol. 32, 56. 10.4014/jmb.2109.09045 34675145 PMC9628830

[B16] Kothari D. Patel S. Kim S. K. (2019). Probiotic supplements might not be universally-effective and safe: a review. Biomed. Pharmacother. 111, 537–547. 10.1016/j.biopha.2018.12.104 30597307

[B17] Li Z. Zhou Y. Li T. Zhang J. Tian H. (2022). Stimuli-responsive hydrogels: fabrication and biomedical applications. View 3, 20200112. 10.1002/VIW.20200112

[B18] Ma T. Zhai X. Jin M. Huang Y. Zhang M. Pan H.. (2022). Multifunctional wound dressing for highly efficient treatment of chronic diabetic wounds. View 3, 20220045. 10.1002/VIW.20220045

[B19] Marco S. Sichetti M. Muradyan D. Piccioni M. Traina G. Pagiotti R.. (2018). Probiotic cell-free supernatants exhibited anti-inflammatory and antioxidant activity on human gut epithelial cells and macrophages stimulated with LPS. Evid. Based Compl. Altern. Med. 2018, 1756308. 10.1155/2018/1756308 PMC6057331 30069221

[B20] Mei L. Zhang D. Shao H. Hao Y. Zhang T. Zheng W.. (2022). Injectable and self-healing probiotics-loaded hydrogel for promoting superbacteria-infected wound healing. ACS Appl. Mater. Interf. 14, 20538–20550. 10.1021/acsami.1c23713 35471815

[B21] Melo T. A. Dos Santos T. F. de Almeida M. E. Junior L. A. G. F. Andrade E. F. Rezende R. P.. (2016). Inhibition of staphylococcus aureus biofilm by *Lactobacillus* isolated from fine cocoa. BMC Microbiol. 16, 1–9. 10.1186/s12866-016-0871-8 27793096 PMC5084336

[B22] Moghadam S. S. Mohammad N. Ghooshchian M. FathiZadeh S. Khodaii Z. Faramarzi M.. (2020). Comparison of the effects of *Lactobacillus plantarum* versus imipenem on infected burn wound healing. Med. J. Islamic Iran 34, 94. 10.34171/MJIRI.34.94 PMC7722975 33315993

[B23] Moreira C. F. Cassini-Vieira P. Canesso M. C. C. Felipetto M. Ranfley H. Teixeira M. M.. (2021). Lactobacillus rhamnosus CGMCC 1.3724 (LPR) improves skin wound healing and reduces scar formation in mice. Prob. Antimic. Proteins 13, 709–719. 10.1007/s12602-020-09713-z 33433898

[B24] Naylor A. R. Hayes P. D. Darke S. (2001). A prospective audit of complex wound and graft infections in great Britain and Ireland: the emergence of MRSA. Eur. J. Vasc. Endov. Surg. 21, 289–294. 10.1053/ejvs.2001.1311 11359327

[B25] Onbas T. Osmanagaoglu O. Kiran F. (2019). Potential properties of lactobacillus plantarum F-10 as a bio-control strategy for wound infections. Probiot. Antimic. Prot. 11, 1110–1123. 10.1007/s12602-018-9486-8 30523603

[B26] Ong J. S. Taylor T. D. Yong C. C. Khoo B. Y. Sasidharan S. Choi S. B.. (2020). Lactobacillus Plantarum USM8613 aids in wound healing and suppresses staphylococcus aureus infection at wound sites. Prob. Antimic. Proteins 12, 125–137. 10.1007/s12602-018-9505-9 30659503

[B27] Parisot J. Carey S. Breukink E. Chan W. C. Narbad A. Bonev B. (2008). Molecular mechanism of target recognition by subtilin, a class I lanthionine antibiotic. Antimic. Agents Chemother. 52, 612–618. 10.1128/AAC.00836-07 PMC2224776 17999970

[B28] Perumal S. Mahmud R. (2013). Chemical analysis, inhibition of biofilm formation and biofilm eradication potential of Euphorbia hirta *L. against* clinical isolates and standard strains. BMC Complem. Alter. Med. 13, 1–8. 10.1186/1472-6882-13-346 PMC4029191 24321370

[B29] Prince T. McBain A. J. O'Neill C. A. (2012). Lactobacillus reuteri protects epidermal keratinocytes from staphylococcus aureus-induced cell death by competitive exclusion. Appl. Environ. Microbiol. 78, 5119. 10.1128/AEM.00595-12 22582077 PMC3416415

[B30] Reddy S. L. C. Grayson A. D. Smith G. Warwick R. Chalmers J. A. C. (2007). Methicillin resistant *Staphylococcus aureus* infections following cardiac surgery: incidence, impact and identifying adverse outcome traits. Eur. J. Cardio-Thor. Surg. 32, 113–117. 10.1016/j.ejcts.2007.03.009 17434315

[B31] Roy R. Tiwari M. Donelli G. Tiwari V. (2018). Strategies for combating bacterial biofilms: a focus on anti-biofilm agents and their mechanisms of action. Virulence 9, 522–554. 10.1080/21505594.2017.1313372 28362216 PMC5955472

[B32] Sancineto L. Piccioni M. De Marco S. Pagiotti R. Nascimento V. Braga A. L.. (2016). Diphenyl diselenide derivatives inhibit microbial biofilm formation involved in wound infection. BMC Microbiol. 16, 1–10. 10.1186/s12866-016-0837-x 27654924 PMC5031294

[B33] Sandhu S. K. Raut J. Kumar S. Singh M. Ahmed B. Singh J.. (2023). Nanocurcumin and viable *Lactobacillus plantarum* based sponge dressing for skin wound healing. Int. J. Pharmac. 643, 123187. 10.1016/j.ijpharm.2023.123187 37394156

[B34] Scriven J. M. Silva P. Swann R. A. Thompson M. M. Naylor A. R. Bell P. R. F.. (2003). The acquisition of methicillin-resistant Staphylococcus aureus (MRSA) in vascular patients. Eur. J. Vasc. Endov. Surg. 25, 147–151. 10.1053/ejvs.2002.1829 12552476

[B35] Siddiqui A. H. Koirala J. (2022). “Methicillin resistant staphylococcus aureus,” in Vulvar Disease: Breaking the Myths 301–2.

[B36] Sikorska H. Smoragiewicz W. (2013). Role of probiotics in the prevention and treatment of meticillin-resistant staphylococcus aureus infections. Int. J. Antimic. Agents 42, 475–481. 10.1016/j.ijantimicag.2013.08.003 24071026

[B37] Simonetti O. Marasca S. Candelora M. Rizzetto G. Radi G. Molinelli E.. (2022). Methicillin-resistant staphylococcus aureus as a cause of chronic wound infections: alternative strategies for management. AIMS Microbiol. 8, 125. 10.3934/microbiol.2022011 35974994 PMC9329881

[B38] Sinha A. Sagar S. Madhumathy M. Osborne W. J. (2019). Probiotic bacteria in wound healing; an *in-vivo* study. Iranian J. Biotechnol. 17, e2188. 10.30498/IJB.2019.85188 PMC7357700 32671124

[B39] Song Y. Sun M. Feng L. Liang X. Song X. Mu G.. (2020). Antibiofilm activity of Lactobacillus plantarum 12 exopolysaccharides against Shigella flexneri. Appl. Environ. Microbiol. 86, e00694–e00620. 10.1128/AEM.00694-20 32444475 PMC7376565

[B40] Sornsenee P. Chatatikun M. Mitsuwan W. Kongpol K. Kooltheat N. Sohbenalee S.. (2021). Lyophilized cell-free supernatants of Lactobacillus isolates exhibited antibiofilm, antioxidant, and reduces nitric oxide activity in lipopolysaccharide-stimulated RAW 264.7 cells. PeerJ. 9, e12586. 10.7717/peerj.12586 34909285 PMC8641486

[B41] Sreevani S. Kumari J. P. (2013). Counter activity of probiotics with reference to pathogenic bacteria. Int. J. Pharm. Bio. Sci. 4, 29–38.

[B42] Srivastava N. Choudhury A. R. (2021). Green synthesis of PH-responsive, self-assembled, novel polysaccharide composite hydrogel and its application in selective capture of cationic/anionic dyes. Front. Chem. 9, 761682. 10.3389/fchem.2021.761682 34778212 PMC8579077

[B43] Stapleton P. D. Taylor P. W. (2002). Methicillin resistance in staphylococcus aureus: mechanisms and modulation. Sci. Progr. 85, 57. 10.3184/003685002783238870 11969119 PMC2065735

[B44] Sürmeli M. Maçin S. Akyön Y. Kayikçioglu A. U. (2019). The protective effect of *Lactobacillus plantarum* against meticillin-resistant staphylococcus aureus infections: an experimental animal model. J. Wound Care 28, S29–34. 10.12968/jowc.2019.28.Sup3b.S29 30840532

[B45] Thimmappa L. Bhat A. Hande M. Mukhopadhyay C. Devi E. Nayak B.. (2021). Risk factors for wound infection caused by Methicillin Resistant *Staphylococcus aureus* among hospitalized patients: a case control study from a tertiary care hospital in India. African Health Sci. 21, 286–294. 10.4314/ahs.v21i1.37 PMC8356623 34394309

[B46] Tong S. Y. Davis J. S. Eichenberger E. Holland T. L. Fowler Jr V. G. (2015). Staphylococcus aureus infections: epidemiology, pathophysiology, clinical manifestations, and management. Clin. Microbiol. Rev. 28, 603–661. 10.1128/CMR.00134-14 26016486 PMC4451395

[B47] von Ossowski I. Pietilä T. E. Rintahaka J. Nummenmaa E. Mäkinen V-M. Reunanen J.. (2013). Using recombinant lactococci as an approach to dissect the immunomodulating capacity of surface piliation in probiotic *Lactobacillus rhamnosus* GG. PLoS ONE 8, e64416. 10.1371/journal.pone.0064416 23691212 PMC3653913

[B48] Xing J. Wang G. Zhang Q. Liu X. Gu Z. Zhang H.. (2015). Determining antioxidant activities of lactobacilli cell-free supernatants by cellular antioxidant assay: a comparison with traditional methods. PLoS ONE 10, e0119058. 10.1371/journal.pone.0119058 25789875 PMC4366247

[B49] Xiu W. Shan J. Yang K. Xiao H. Yuwen L. Wang L. (2021). Recent development of nanomedicine for the treatment of bacterial biofilm infections. View 2, 20200065. 10.1002/VIW.20200065

[B50] Yang F. Liu C. Ji J. Cao W. Ding B. Xu X. (2021). Molecular characteristics, antimicrobial resistance, and biofilm formation of *Pseudomonas aeruginosa* isolated from patients with aural infections in Shanghai, China. Infect. Drug Resist. 14, 3637–3645. 10.2147/IDR.S328781 34522106 PMC8434892

[B51] Zhong D. Du Z. Zhou M. (2021). Algae: a natural active material for biomedical applications. View 2, 20200189. 10.1002/VIW.20200189

